# Elucidating the Mechanism of Buyanghuanwu Decoction Acting on Pulmonary Fibrosis Based on Network Pharmacology and Animal Studies

**DOI:** 10.2174/1386207326666230823093958

**Published:** 2024-04-26

**Authors:** Qichang Xing, Xiang Liu, Zheng Liu, Qingzi Yan, Yixiang Hu, Wencan Li, Ke Peng

**Affiliations:** 1 Department of Clinical Pharmacy, Xiangtan Central Hospital, Xiangtan, Hunan, 411100, China;; 2 Zhou Honghao Research Institute Xiangtan, Xiangtan, Hunan, 411100, China;; 3 College of Pharmacy, Changsha Medical College, Changsha, Hunan, 410219, China

**Keywords:** Buyanghuanwu decoction, pulmonary fibrosis, network pharmacology, TCMSP, bleomycin, KEGG

## Abstract

**Background and Objective:**

Buyanghuanwu Decoction (BYHWD) is a clinically proven prescription effective in treating pulmonary fibrosis (PF), but the molecular mechanism underlying its action remains unclear. The network pharmacology analysis was performed to elucidate the acting substances and related pathways of BYHWD in treating bleomycin (BLM) induced PF mouse.

**Methods:**

First, the pharmacologically active components and corresponding targets in BYHWD were identified through the TCMSP database and literature review. Second, PF-related targets were identified through the DisGeNet database. Then, the components-targets network of BYHWD in PF treatment was constructed using Cytoscape. The DAVID database was used for the enrichment analysis of GO terms and KEGG pathways. At last, the therapeutic effect of BYHWD on BLM-induced PF mice were verified, and the mRNA and protein expression of related targets was determined through RT-PCR and western blotting, respectively.

**Results:**

The core component-target network contained 58 active components and 147 targets. Thirty-nine core targets were mainly involved in the regulation of biological functions and KEGG pathways, such as the positive regulation of nitric oxide biosynthesis and the TNF signaling pathway. These core targets were obtained through enrichment analysis. Moreover, animal studies revealed that BYHWD down-regulated the mRNA expression levels of TNF, IL-6, IL-1β, and NOS2 and inhibited NF-κB and p38 phosphorylation.

**Conclusion:**

The effects of BYHWD on PF mice are therapeutic, and its anti-PF mechanism mainly involves the effects on inflammatory factors and the NF-κB/p38 pathway.

## 1 INTRODUCTION

Pulmonary fibrosis (PF) is a chronic and life-threatening disease that involves a progressive decline in lung function. The pathological features of PF are conjectured to be due to the excessive deposition of dysregulated extracellular matrix (ECM) proteins leading to fibroblast proliferation and abnormal re-epithelialization, following alveolar injury and progressive respiratory failure [1]. PF can be idiopathic (idiopathic pulmonary fibrosis, IPF) or secondary to various medical conditions. In recent years, the incidence of PF has increased significantly worldwide [2]. Because of its hidden onset and no specific clinical manifestations, PF diagnosis is difficult at an early stage. In addition, no special treatment is currently available for PF, and PF-related mortality is very high. The average survival time after PF diagnosis is 2-4 years, with the 5-year survival rate being only 30% ~ 50% [3], PF has become a serious health concern, and therefore, research on the mechanism of PF and drugs effective against PF holds important scientific value. Buyanghuanwu Decoction (BYHWD) is an effective prescription for PF treatment, which was created by Wang Qingren, a famous physician of traditional Chinese medicine in the Qing Dynasty [4]. BYHWD is composed of *Astragalus membranaceus* (Fisch.) Bunge (Huang qi), *Angelica sinensis* (Oliv.) Diels (Dang gui), *Prunus persica* (L.) Batsch (Tao ren), *Pheretima aspergillum* (E. Perrier) (Di long), *Ligusticum striatum* DC. (Chuan xiong), *Carthamus tinctorius* L. (Hong hua), and *Radix Paeoniae Rubra* (Chi shao). In this formula, drugs are compatible with each other and coordinate to achieve the effects of tonifying qi, activating blood circulation and dredging collaterals. According to modern pharmacological studies, BYHWD experts have pharmacological effects such as antithrombosis, improving hemorheology, scavenging free radicals, and antioxidation [5]. Animal studies have found that BYHWD can improve the pulmonary function index of PF rats [6]. Therefore, this study used network pharmacology technology to determine the interventional effect of BYHWD on PF mice, and thus provide an experimental basis for the pharmacodynamic material basis and action mechanism of BYHWD.

## 2 MATERIALS AND METHODS

### 2.1 Collection of the Active BYHWD Components and Screening Potential Targets

The components of Huangqi, Danggui, Honghua, Taoren, Chishao, and Chuanxiong were obtained through the Traditional Chinese Medicine Systems Pharmacology Database (TCMSP, https://old.tcmsp-e.com/tcmsp.php) [7] and the components of Dilong were collected from literature [8]. According to the relevant parameters of the pharmacokinetic properties of the component, the active BYHWD components were screened on the basis of oral bioavailability (OB) ≥30% and drug-likeness (DL) ≥0.18. Then, the targets of the active components were collected from TCMSP. Finally, Uniprot (http://Uniprot.org) was used to convert all the names of the target to respective gene symbols.

### 2.2 Screening the Potential Targets from BYHWD and Meanwhile Related to PF

PF-related targets were collected from the DisGeNET database (https://www.disgenet.org/) [9] by using “idiopathic pulmonary fibrosis” as the keyword. The BYHWD active component targets and PF-related targets were intersected to obtain potential key targets for BYHWD for PF treatment. The BYHWD active components targets and PF-related targets were imported into VENNY 2.1 (https://bioinfogp.cnb.csic.es/tools/venny/) for visualization.

### 2.3 Visualization of the Herbs-components-targets Network

The relationships between the active components and predicted key targets, as well as those between the herbs and active components, were clarified using the herb-component-target interaction network, which was visualized and constructed using Cytoscape 3.9.0 software [10].

### 2.4 Construction of Protein-protein Interaction Network

To construct the protein-protein interaction (PPI) network, we input the potential key targets into the search tool for the STRING database (https://www.string-db.org/) [11]. The organism was set as Homo Sapiens. The minimum required interaction score was established as 0.9.

### 2.5 GO and KEGG Enrichment Analyses

The potential key targets were identified using Database for Annotation, Visualization and Integrated Discovery (DAVID) v6.8 (https://david.ncifcrf.gov/) [12]. A threshold of *P*<0.05 was used to identify core GO and KEGG pathways.

### 2.6 Preparation of BYHWD

BYHWD is composed of Huangqi, Danggui, Chishao, Dilong, Chuanxiong, Honghua and Taoren at 120:6:4.5:3:3:3:3 ratio (dry weight) and was purchased from the Xiangtan Central Hospital (Xiangtan, China). All herbs were mixed and extracted through heating and refluxing twice with 10 volumes of 60% ethanol. The filtrates were combined and concentrated to 2 g/mL (according to the raw herbs).

### 2.7 Experimental Animals

In total, 40 male ICR mice (weight 21-25 g, age 6-8 weeks) were purchased from Nanjing Cavens Biotechnology Co., Ltd. (Certificate No. SCXK [Hu] 2018-0006, Shanghai, China). All mice were maintained in a controlled environment at a 12 h/12 h light/dark cycle and 22°C±2°C with 55%±2% humidity. The animals had free access to food and water. Animal welfare and the experimental procedures were in accordance with the Care and Use of Laboratory Animals and were approved by the Ethical Committee of Chang-sha Medical University.

### 2.8 Establishment of BLM-induced PF and Treatment with BYHWD

All mice were fed adaptively for 1 week and then randomly divided into two groups: Sham group (n = 10), and Model group (n = 30). The Model group was given tracheal instillation of BLM at 2.0 mg/kg body mass on day 0 [13]. Then, the Model group mice were divided into three groups on the basis of weight: BLM group (n = 10), BLM+BYHWD (18.5 g/kg) (n = 10), BLM+BYHWD (9.25 g/kg) (n = 10). A single dose of BYHWD was orally administered to the BLM+BYHWD groups on day 3. The Sham and BLM groups were given an equal volume of vehicles (0.9% NaCl). On day 17, all mice were intraperitoneally anesthetized with 20% urethane (1000 mg/kg) and their lungs were harvested and weighed. The left lungs were collected for histopathological examination, while the right lungs were frozen in liquid nitrogen for quantitative real-time (RT)-PCR, hydroxyproline (HYP) determination, and western blotting.

### 2.9 Pathological Examination

To observe pathological changes and examine the fibrotic extent, the left lung samples were fixed in 10% formalin for 48 h, dehydrated with graded ethanol, embedded in paraffin, cut into 4 μm thick lung sections and stained with hematoxylin-eosin (H&E) and Masson. The severity of alveolitis and fibrosis was subsequently scored by referring to Ashcroft. The Ashcroft scoring criteria were as follows [14]: normal lung or minimal fibrous thickening of alveolar or bronchiolar walls, 0-1 points; moderate thickening of these walls without any obvious damage to the lung architecture, 2-3 points; increased fibrosis with definite damage to the lung structure and formation of fibrous bands or small fibrous masses, 4-5 points; severe distortion of the lung structure and large fibrous areas; a “honeycomb lung” is included in this category, 6-7 points; total fibrous obliteration of the field, 8 points.

### 2.10 HYP Content Determination

HYP, a product of collagen hydrolysis, is considered among the markers of fibrosis. HYP was determined through the alkaline hydrolysis method by using an HYP assay kit (Nanjing Jiancheng Bioengineering Institute). About 30-100 mg of lung tissue was weighed, and 1 mL of alkaline hydrolysis solution from the kit was added to the tissue sample. The tissue suspension was heated and hydrolyzed in a water bath at 95°C for 20 min, and after hydrolysis was completed, 10 mL of pH indicator was added. The pH value was adjusted with the basic conditioning solution in the kit to allow the mixture to turn red and become red. Then, using the acidic conditioning solution in the kit, the pH value was adjusted to 7.0. At pH 7.0, the red solution turned yellow-green, and 10 mL of double-distilled water (ddH_2_O) was added to the solution. Then, 30 mg activated carbon was added to 4 mL diluted hydrolysate, mixed, and centrifuged at 3500 rpm/min for 10 min at 4 °C. The supernatant was used for testing. Then, assay reagents 1, 2, and 3 in the kit were added according to the reaction and reaction temperature specified in the instructions of the kit. The reaction supernatant was used for measuring the absorbance at 550 nm. The HYP content was calculated using the absorbance values of the blank, standard, and sample wells.

### 2.11 RNA Isolation and Real-time PCR

The lung samples (approximately 20 mg) were cut, weighed, and homogenized using a tissue homogenizer in 500 μL buffer RL (FastPure® Cell/Tissue Total RNA Isolation Kit V2) to isolate RNA. Then, the samples were centrifuged at 12000 rpm/min and 4°C for 10 min. The supernatant was added to the DNA adsorption column, and centrifuged. The filtrate was collected and dissolved in 250 μL anhydrous ethanol. The mixed solution was transferred to the RNA adsorption column. The solution was washed once with the deproteinized solution and twice with a washing solution. After the adsorption column was air-dried, 30 μL non-nuclease dd-H_2_O (Dnase and Rnase-free dd-H_2_O) was added to each sample to elute RNA. A nanodrop spectrophotometer (Thermo Scientific) was used to determine the RNA concentration. cDNA was synthesized from 1 μg isolated RNA by using 4 μL HiScript Q RT SuperMix (Vazyme, Nanjing) according to the manufacturer’s instructions. qRT-PCR (20 μL reaction volume with 1μL cDNA, 0.4 μL each gene-specific primer and FastStart Universal SYBR Green Master (ROX) (Roche) 10 uL, 0.2 μL non-nuclease dd-H_2_O) were performed using RT-qPCR QuantStudio 3(Life technologies). PCR amplifications were performed at 95°C for 30s for denaturation and 40 cycles of annealing/elongation (60°C, 30s) for selected genes (Table **1**). RT-PCR primers were designed using Primer5 software. Gene expression was normalized to the expression of the house-keeping gene GAPDH and quantified using the 2^−ΔΔCt^ method to relatively quantify the expression of genes of our interest.

### 2.12 Western Blotting

Lung tissue samples were collected and lysed on ice with ice-cold RIPA buffer (Beijing Dingguo Changsheng Biotechnology Co., Ltd) containing a protease inhibitor, and PMSF, for 30 min. This was followed by centrifugation (12,000 *g*/min, 15 min). The protein concentration in the supernatants was determined using the BCA protein assay kit (Beijing Dingguo Changsheng Biotechnology Co., Ltd). The loading buffer was mixed in the supernatants at 1: 4 ratio and placed in boiling water for 10 minutes. Protein (20 µg) was separated using 8% SDS-PAGE gel and transferred onto nitrocellulose membranes. The blotted membranes were blocked with 5% non-fat dairy milk and incubated overnight at 4°C with the indicated primary antibodies, including anti-rabit NF-κB (p65) (Affinity Biosciences, 1:1000, AF5006), p-p65 (Affinity Biosciences, 1:1000, AF2006), p38 mitogen-activated protein kinase (MAPK) (ABclonal Technology Co., Ltd., 1:1000, A14401), p-p38 kinase (ABclonal Technology Co., Ltd., 1:1000, AP0057), and GAPDH (ABclonal Technology Co., Ltd., 1:10000, AC002). The membranes were subsequently incubated with secondary antibodies for 1 h at room temperature. Finally, western blotting was visualized using the ECL reagent (Gen-View Scientific Inc.) and analyzed using the Gel Image System (Ver.4.00).

### 2.13 Statistical Analysis

All quantitative data are expressed as the means ± standard deviation (SD). Statistical differences between the groups were evaluated through one-way analysis of variance (ANOVA) with Dunnett’s multiple comparison tests by using GraphPad Prism software (version 8.0; San Diego, California USA, www.graphpad.com). The statistical significance was established at a *P* value <0.05.

## 3 RESULTS

### 3.1 Screening of Potential Pharmacodynamic Components and Targets of BYHWD

In total, 669 chemical components were identified in the six herbs from the TCMSP database, and 83 chemical components in the Dilong through literature search (Supplementary Table **S1**). Thereafter, all components were screened at the active ingredient thresholds of OB≥30% and DL≥0.18. A total of 92 potential components were obtained. The targets of these 92 components were obtained by searching the TCMSP databases. At last, 58 components that corresponded to 147 targets were collected. The illustration of drug-component corresponding relationship was shown in the Fig. (**1A**).

### 3.2 Mining of Potential Core Targets of BYHWD for PF Treatment

In total, 764 PF-related targets were obtained through retrieval from the DisGeNET database. Subsequently, as shown in Fig. (**1B**), the intersection of the 147 BYHWD components corresponded targets and the 764 PF-related target genes were obtained using Venn diagrams. Thirty-nine intersected targets were considered the potential core targets of BYHWD for PF treatment.

### 3.3 Construction of the “Herb-Component-Target” and PPI Networks

**Fig. (2):**
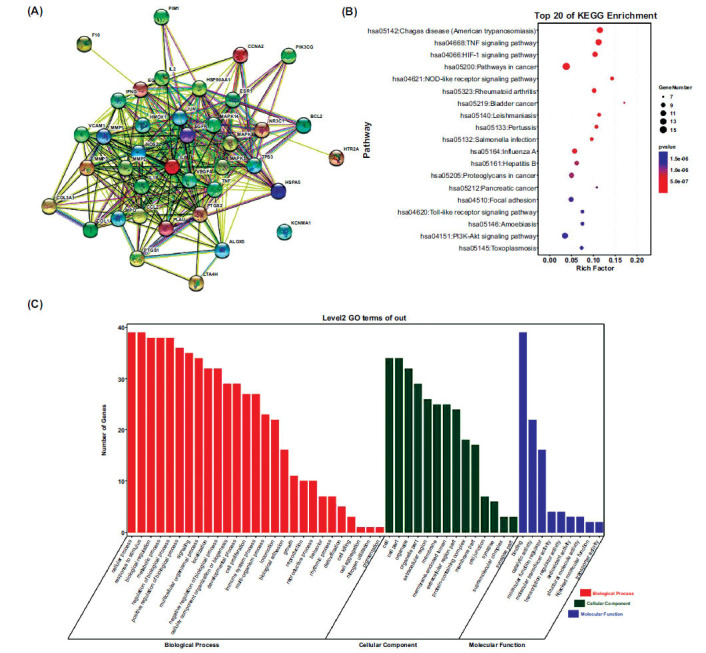
KEGG pathway and GO enrichment analysis. (**A**) Protein-Protein Interaction Networks. (**B**) Top 20 of pathway enrichment. (**C**) GO second class enrichment analysis of 39 potential targets related to PF.

The candidate component-target network of BYHWD for PF treatment was constructed using Cytoscape software. As shown in Fig. (**1C**), 97 nodes and 336 edges were contained in the network and the mean degree of candidate components was 6.93. The degree value of 24 ingredients was higher than 6.93. The 10 active ingredients with the highest degree value were presented in Table **2**. In particular, quercetin, luteolin, and kaempferol acted on 34, 21 and 17 targets, respectively, and thus are representative active components of BYHWD. Regarding targets, ESR1, PTGS2, NOS2, and PTGS1 were targeted by 46, 42, 32, and 27 of BYHWD components, respectively, suggesting that these targets are mainly involved in the underlying action mechanisms BYHWD. The PPI analysis was performed on 39 potential core targets (Fig. **2A**). The degree value represents the centrality of the target (Supplementary Table **S4**). TNF, IL6, IL1B and NOS2 were highly expressed in the lung tissue and inflammatory cells and were speculated as the core targets. This was validated through PCR experiments (Supplementary Table **S5**).

### 3.4 Enrichment Analysis of the Core Targets

The possible action mechanisms of BYHWD in PF treatment were explored by performing the GO and KEGG pathway enrichment analyses on the 39 core targets. In total, 239 Biological Processes (BP), 29 Cell Components (CC), and 41 Molecular Function (MF) were obtained in the GO analysis (Supplementary Table **S2**). The top 27 terms in BPs, 14 terms in MFs, and 9 terms in CCs are presented in Fig. (**2C**). BYHWD was suggested to attenuate PF possibly through the positive regulation of nitric oxide biosynthesis, cellular response to lipopolysaccharides, *etc*. More than 93 pathways, including Chagas disease (American trypanosomiasis), TNF signaling pathway, HIF-1 signaling pathway, Pathways in cancer, NOD-like receptor signaling pathway were identified in the KEGG enrichment analysis (Supplementary Table **S3**). The top 20 KEGG pathways were selected based on their *p*-values to generate a bubble chart for visualization (Fig. **2B**).

### 3.5 Anti-PF Effects of BYHWD in the BLM-induced Mouse Model

**Fig. (3):**
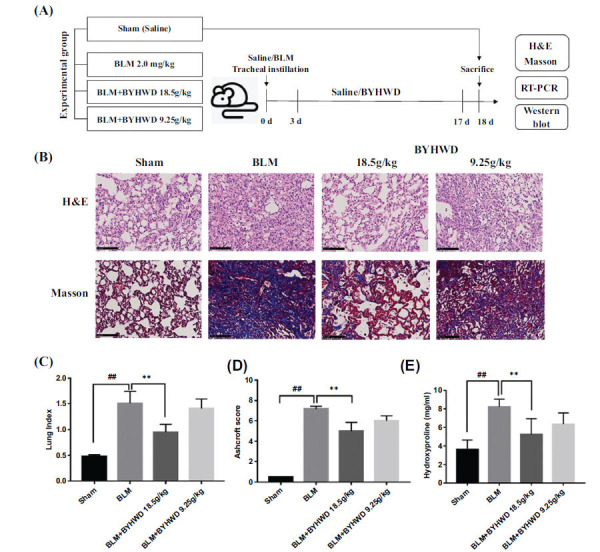
Effects of BYHWD on PF in BLM-induced mice model. (**A**) Flow chart of animal experiment. (**B**) H&E staining and Masson’s trichrome staining. Original magnification 200×, Scale bars = 100 μm. (**C**) Comparison of the lung index among the experimental groups. (**D**) Comparison of the Ashcroft score among the experimental groups. (**E**) Comparison of the HYP content among the experimental groups. ##*P* < 0.05 BLM group *vs*. Sham control group, **P < 0.01 BLM group *vs*. BYHWD-treated group 18.5g/kg.

In order to validate the therapeutic effect of BYHWD, the PF micemodel was established (Fig. **3A**). The H&E staining assessed pathological changes in lung tissues. The Sham group exhibited no obvious degeneration and shedding of the bronchial epithelium, no collapse of the alveolar wall and emphysema, no obvious disappearance of the alveolar structure, a small amount of chronic inflammatory cell infiltration in the alveolar septum, and no clear fibrous tissue hyperplasia. The alveolar structure in the BLM group was severely damaged and exhibited degeneration and exfoliation of the bronchial epithelium, alveolar wall collapse with mild emphysema, disappearance of the normal structure of part of the alveolar cavity with fibrous tissue proliferation and infiltration of several inflammatory cells, and eosinophil accumulation around the tube (Fig. **3B**). Compared with the BLM group, pathological changes in the BYHWD-treated group were improved to different degrees. Moreover, the lung tissue structure in the Sham group was intact with a thin collagenous layer of the bronchus wall, as observed in Masson’s staining. The alveolar structure in the BLM group was seriously damaged compared with the Sham group. The number of collagen fibers in the bronchial wall, lung interstitium and alveolar septum was significantly increased, indicating that BLM induced severe PF (Fig. **3B**).The lung index is the lung weight to body weight ratio. This index is among the indicators of the degree of inflammation and fibrosis in the lung tissue. During the formation of interstitial lung fibrosis, the initial increased lung weight due to inflammatory exudation, cell swelling and capillary congestion is the early cause of the increased lung coefficient. With a gradual decrease in inflammation, the deposition of collagen fibers increases, leading to interstitial fibrosis. The hardening of lung tissue, pallor, reduced elasticity, and increased lung weight are the late causes of the increased lung index. The lung index of the BLM group was significantly increased compared with that of the Sham group (*P*< 0.01), and the BYHWD-treated groups presented a significant decrease in the lung index compared with the BLM group (Fig. **3C**). Furthermore, the Ashcroft score and HYP content of the BLM group was significantly higher than that of the Sham group(*P*< 0.01; Fig. **3D** and **3E**). Compared with the BLM group, the Ashcroft scores and HYP content of the BYHWD-treated groups improved to different degrees.

### 3.6 Validation of Predicted Target Through RT-PCR

TNF-α directly stimulates the proliferation of cultured lung fibroblasts, as well as induces them to release IL-6. TNF-α, and IL-6 jointly promote PF. IL-1β is another crucial inflammatory factor that causes fibrosis and is the main inducer of the pro-inflammatory response. iNOS is a type of nitric oxide synthase (NOS). iNOS is barely expressed in cells of various tissues under normal circumstances but is activated when some cytokines are induced under pathological conditions. This leads to an increase in NO synthesis, thus involved in angiogenesis, tumor progression, and many other pathological processes. As shown in Fig. (**4**), BYHWD administration reduced the mRNA expression of predicted targets, including TNF, IL-6, IL-1β, and NOS2, compared with that in the BLM-induced PF mice.

### 3.7 BYHWD Administration Inhibited NF-κB/p38 Phosphorylation

The MAPK cascade signaling pathway is among the most critical intracellular signaling pathways, mainly divided into ERK, JNK, and p38 MAPK pathways, involved in different biological processes. Among them, the p38 MAPK pathway is mainly closely related to inflammation, cell growth and stress response. p38 is the upstream gene of NF-κB, a crucial intracellular nuclear transcription factor. NF-κB is involved in the transcriptional regulation of various cytokines and inflammatory mediators. It plays a critical role in the regulation of the inflammatory response network. NF-κB/p38 signaling molecules play a crucial role in PF. We here examined the effect of BYHWD on NF-κB and p38 activation through western blotting, as shown in Fig. (**5**). Treatment with BYHWD significantly inhibited p65 NF-κB and p38 MAPK phosphorylation in BLM-challenged lungs.

## 4 DISCUSSION

PF is a refractory and complex disease. Currently, anti-inflammatory drugs, immunosuppressants and anti-fibrosis drugs are primarily used for PF treatment, but their clinical efficacy is poor, possibly because of the single target of these drugs.

In recent years, an increasing number of scholars have clearly realized the need to develop multi-target and multi-pathway drugs to treat this chronic and complex disease. The active components in the Traditional Chinese Medicine (TCM) component formulas exert a synergistic therapeutic effect by acting on multiple targets. Studies on the multiple links and multi-target system modulating effects of the aforementioned active components will be a critical future research direction for understanding the action mechanism of herbal medicines [15].

Herbal components and single herbal extracts have a certain influence on PF [16-18]. The 1994 trial guidelines for PF diagnosis and treatment recommended TCM therapy as an experimental therapy [19]. TCM is a reflection of Chinese people's long-term struggle against disease. TCM is easily accepted in China, and almost all PF patients received TCM treatment. In TCM, PF belongs to the categories of “feiwei”(which means pulmonary fistula) and “feibi” (which means pulmonary arthralgia) [20]. PF is mostly responsible for the deficiency of lung and kidney Qi, the entry of evil Qi, the prolonged retention of lung collaterals, Qi and blood stagnation due to blood stasis, and flaccid lung collaterals due to arthralgia. Therefore, Qi deficiency and blood stasis are the main PF pathogenesis, and supplementing Qi and activating blood circulation are the basic methods of treating this disease [21]. Here, based on the clinical observations and TCM theory of “supplementing Qi and activating blood circulation”, we proposed that BYHWD could be an effective treatment for PF. In the BLM-induced PF model, the mice exhibited weight loss and reduced activity after 3 days of BLM action, which is consistent with the evidence of qi deficiency in TCM. Our previous study also found that a 20% weight loss after 3 days of BLM induction is likely to progress to PF after 14 days. Therefore, we chose to administer the BYHWD on day 3 after BLM induction in this experiment to observe the therapeutic effect of BYHWD on PF mice with Qi deficiency and blood stasis. Our results also confirmed that BYHWD could improve pathological changes in the mice with BLM-induced PF.

Meanwhile, we attempted to explain the molecular mechanism of BYHWD against PF by using the network pharmacology method. The major findings were as follows: according to the drug-component-targer network, 58 components corresponding to 147 targets were considered as potential components and targets associated with BYHWD against PF; quercetin, luteolin, and kaempferol were probably the key BYHWD components involved in PF treatment because they affected most PF-related targets; and KEGG and GO enrichment analyses revealed that BYHWD was involved in the TNF signaling pathway. BYHWD was also involved in 239 BPs, 29 CCs, and 41 MFs, including the cellular process, cell part, and binding function; The top 10 proteins in the center of the PPI network, namely TNF, IL6, TP53, VEGFA, IL1B, JUN, PTGS2, CCL2, EGF, and EGFR. These proteins were the core targets that play a major role in PF treatment. These findings revealed the “multi-ingredient, multi-target, multi-pathway” characteristics of BYHWD, and explained the synergistic regulatory effect of each BYHWD component from the overall viewpoint. Quercetin is a type of flavonol component with various biological activities, such as anti-inflammatory, anti-oxidant, anti-PF, and anti-cancer activities [22-24]. Luteolin is a natural flavonoid present in many plants and exhibits strong anti-inflammatory activity *in vitro* and *in vivo* [25]. Kaempferol could alleviate silica-induced PF by modulating autophagy [26].

Current studies have shown that PF is closely related to the inflammatory process [27]. Aggregation of inflammatory cells and cytokines release induce numerous collagen fibers, resulting in the remodeling of the lung tissue structure, reduction in alveolar number, deformation, atresia, and loss of lung function [28]. TNF and HIF-1 pathways are closely related to the inflammatory response and interfere with PF occurrence and development, which is consistent with the present study results [29, 30]. TNF-α plays multiple roles in fibrotic pathology by inducing the accumulation of inflammatory cells and factors, such as TGF-beta, IL-1, and IL-6, thereby promoting myofibroblast differentiation to synthesize ECM, and causing tissue remodeling [31]. Nintedanib inhibits PF by reducing TNF-α and IL-6 expression and decreasing alveolar wall thickening [32]. IL-1β, which can be secreted and produced by various activated immune cells, is an important player as a powerful immune regulator and pro-inflammatory factor in acute and chronic inflammation [33]. Higher IL-1β levels can be detected in rat models of PF and bronchoalveolar lavage fluid from PF patients [34]. NOSs play different roles in PF [35]. NO concentration in the lung may be critical for maintaining respiratory homeostasis [36]. Strong reductions or elevations in NO levels in lung tissues have been proposed to promote PF. Knockout or pharmacologic inhibition of iNOS was found to attenuate PF. Remarkably, NF-κB and MAPK signaling pathways are indispensable in producing cytokines TNF-α, IL-6, and IL-1β. Inhibitors of the MAPK signaling pathway can down-regulate the expression of these factors. Western blotting revealed that BYHWD decreased p-p38 MAPK and p-p65 NF-κB expression in the lung of the BLM-induced PF mice, suggesting that BYHWD suppresses inflammation and PF by inhibiting the production of inflammatory cytokines through the NF-κB/p38 pathway.

## CONCLUSION

We have analyzed the molecular action mechanism of BYHWD in PF treatment by using the network pharmacology method. We predicted that TNF-α, IL-6, IL-1β, and NOS2 are the crucial targets. These targets may cause internal reactions in the body through multiple pathways such as the NF-κB/p38 pathway, and thus participate in PF development. This study only verified the expression of some inflammatory factors in the core targets. The specific regulatory mechanism needs to be further explored and verified.

## Figures and Tables

**Fig. (1):**
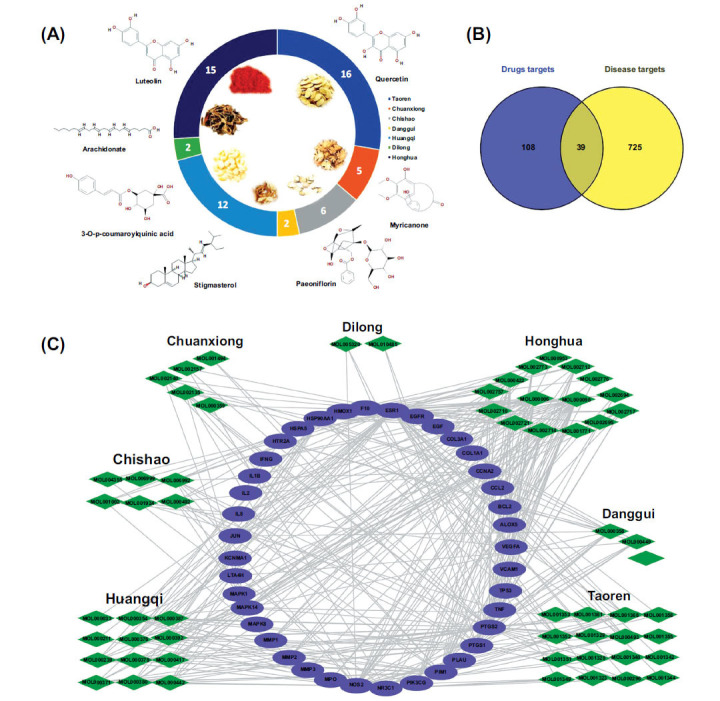
Building a visualization network for BYHWD treatment of PF through network pharmacology. (**A**) Illustration of drug-component corresponding relationship: The 58 active components in BYHWD were derived from 7 drugs. (**B**) Venn diagram depicting the intersection of BYHWD targets and PF targets. The purple circle represents the BYHWD target genes, the yellow circle represents the PF target genes, and the overlap of the two circles indicates BYHWD target genes prediction for PF treatment. (**C**) Drugs-Component-Target-Disease network: The purple nodes represent targets, the green nodes represent targets, and the lines between the nodes represent the interactions between the component and the target.

**Fig. (4):**
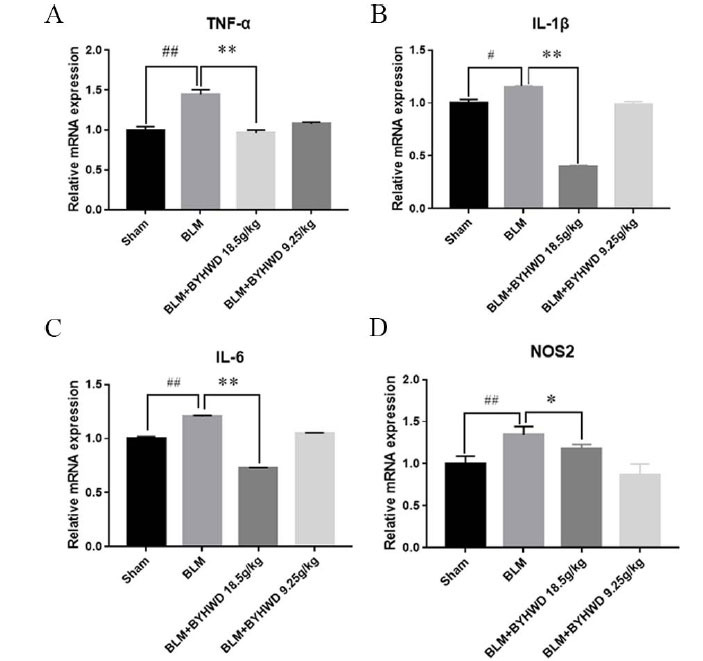
The core targets mRNAs expression was decreased by BYHWD treatment. (**A**) TNF-α, (**B**) IL-6, (**C**) NOS2, and (**D**) IL-1β at the transcriptional level were analyzed in PF mice with or without BYHWD treatment. Data are presented as the means ± SD based on three independent experiments, ##*P* < 0.05 BLM group *vs*. Sham control group, **P< 0.01 BLM group *vs*. BYHWD-treated group 18.5g/kg.

**Fig. (5):**
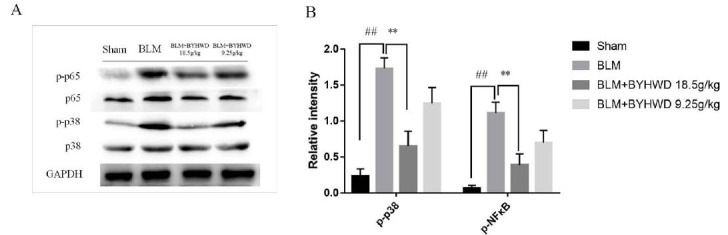
The effect of BYHWD on BLM-stimulated p38 and NF-κB(p65) phosphorylation in the lungs of mice. (**A**) Lung tissue homogenate was prepared, and the relative protein levels of p38, p-p38, p65 and p-p65 were detected by Western blotting, with GAPDH as an internal control. (**B**) Densitometric analysis of the p-p38/p38 and p-p65/p65 ratios was performed using Gel Image System. Data are presented as the means ± SD based on three independent experiments, ##*p* < 0.05 BLM group *vs*. Sham control group, **p < 0.01 BLM group *vs*. BYHWD-treated group 18.5g/kg.

**Table 1:** The primer sequences for target genes in real-time PCR assays.

**Gene**	**5’-3’**
TNF forward TNF reverse	ACTGAACTTCGGGGTGATCG CCACTTGGTGGTTTGTGAGTG
IL-1β forward IL-1β reverse	GCCACCTTTTGACAGTGATGAG TGATGTGCTGCTGCGAGATT
IL-6 forward IL-6 reverse	TGATGGATGCTACCAAACTGG TGTGACTCCAGCTTATCTCTTGG
NOS2 forward NOS2 reverse	ACAGGGAGAAAGCGCAAAAC CCAGGGATTCTGGAACATTCTGT
GAPDH forward GAPDH reverse	TCAGGAGAGTGTTTCCTCGTC CCGTTGAATTTGCCGTGAGT

**Table 2:** Top 10 basic information of ingredients and the targets by their degree values.

**TCMSP ID**	**Chemical Name (Drug)**	**Degree**	**Target**	**Degree**
MOL000098	Quercetin (Huangqi)	34	ESR1	46
MOL000006	Luteolin (Honghua)	21	PTGS2	42
MOL000422	Kaempferol (Honghua)	17	NOS2	32
MOL000358	beta-sitosterol (Danggui)	13	PTGS1	27
MOL002714	Baicalein (Honghua)	13	HSP90AA1	24
MOL000378	7-O-methylisomucronulatol (Huangqi)	11	PIM1	24
MOL000493	Campesterol (Taoren)	9	CCNA2	23
MOL001368	3-O-p-coumaroylquinic acid (Taoren)	9	MAPK14	23
MOL000392	Formononetin (Huangqi)	9	NR3C1	11
MOL000354	Isorhamnetin (Huangqi)	9	PIK3CG	10

## Data Availability

The data that support the findings of this study are available from the corresponding authors [X.L. and Q.X.], upon reasonable request.

## LIST OF ABBREVIATIONS

PF: Pulmonary Fibrosis
BYHWD: Buyanghuanwu Decotion
ECM: Extracellular Matrix
DL: Drug-Likeness
OB: Oral Bioavailability
PPI: Protein-Protein Interactions
GO: Gene Ontology
KEGG: Kyoto Encyclopedia of Genes and Genomes
